# Personal Drug Selection: Problem-Based Learning in Pharmacology: Experience from a Medical School in Nepal

**DOI:** 10.1371/journal.pone.0000524

**Published:** 2007-06-13

**Authors:** P. Ravi Shankar, Subish Palaian, Sudesh Gyawali, Pranaya Mishra, Lalit Mohan

**Affiliations:** Department of Pharmacology, Manipal College of Medical Sciences, Pokhara, Nepal; Instituto de Medicina Tropical Alexander Von Humboldt, Peru

## Abstract

**Background:**

At the Manipal College of Medical Sciences, Pokhara, Nepal, Pharmacology is taught during the first four semesters of the undergraduate medical course. Personal or P-drug selection is an important exercise. The present study was carried out to obtain student opinion about the P-drug learning sessions, the assessment examinations, and on the small group dynamics.

**Method:**

The practical sessions on P-drug selection are carried out in small groups. Student feedback about the session was obtained using focus group discussions. The focus groups were selected to represent both genders and the three main nationalities, Nepalese, Indians, and Sri Lankans. There were four Nepalese, five Indians, and three Sri Lankans. Within each nationality and gender category the students were randomly selected. The respondents were explained the objectives of the study and were invited to participate. Written informed consent was obtained. The discussion lasted around two hours and was conducted in the afternoon in two groups of six students each. The first author (PRS) acted as a facilitator. The responses were recorded and analyzed qualitatively.

**Results:**

The overall student opinion was positive. Around 25% (3 respondents) of respondents were confused about whether P-drugs were for a disease or a patient. Group consensus was commonly used to give numerical values for the different criteria. The large number of brands created problems in calculating cost. The students wanted more time for the exercise in the examination. Formative assessment during the learning sessions may be considered. The group members usually got along well. Absenteeism was a problem and not all members put in their full effort. The physical working environment should be improved.

**Conclusions:**

Based on what the students say, the sessions on P-drugs should be continued and strengthened. Modifications in the sessions are required. Sessions during the clinical years and internship training can be considered.

## Introduction

Traditional teaching in pharmacology was characterized by passive transfer and memorizing of information about drug classes and individual compounds [1]. Medical science in general and therapeutics in particular is undergoing rapid change and an information explosion and it is important to train doctors for self-directed learning [2]. Learning how to evaluate and analyze information is becoming an important skill. Solving problems in therapeutics, prescribing appropriate drugs for a disease condition and delivering drug- and disease-related information in a meaningful way to patients should be regarded as key ‘transferable skills’ in Pharmacology [3].

Irrational prescribing is a common problem [4] and has been referred to as a ‘habit which is difficult to cure’ [5]. Traditional teaching in medical schools does not prepare students for rational therapeutics. A survey in a medical school in the United Kingdom (UK) had revealed that medical students felt the need for more teaching of therapeutics in the undergraduate medical curriculum [6]. Medical schools till recently used to spend less than 1% of the total teaching time on prescribing issues [7].

A method of orientating students towards therapeutics is to expose them to a sequential decision-making process for solving therapeutic problems [8]. In 1994, a manual on the principles of rational prescribing called ‘Guide to Good Prescribing’ was developed by the World Health Organization (WHO) Action Program on Essential Drugs [9]. In 2001, ‘Teachers' Guide to Good Prescribing’ was developed as a companion volume to help medical teachers better use the ‘Guide to Good Prescribing’ to teach undergraduate medical students [10]. These manuals present students with a normative model for pharmacotherapeutic reasoning. Students are taught to develop a standard treatment for common disorders and a set of first-choice drugs called Personal or P-drugs. Students develop their set of P-drugs using National and International treatment guidelines, formularies, textbooks and other sources of drug information [10]. A six-step problem solving approach is used to apply this set of P-drugs to specific patient problems.

The practical exercise on P-drug selection has been carried out for over two years at our institution. We follow the method described by Joshi and Jayawickramarajah [8]. The four criteria of efficacy, safety, cost and convenience are considered while selecting a P-Drug. For a particular disease, each of the four criteria is given a score between 0 and 1 depending on the importance of the criteria for the disease condition. This is termed the weight. The total scores of the four criteria should add up to one. For each drug group/drug a score is given between 1 and 10 for each of the four criteria with a higher score indicating a better value.

The method followed is a modification of the multi-attributive utility analysis (MAUA) described in ‘Teacher's guide to good prescribing’ [10]. First a group is chosen according to the defined criteria and then an individual drug is chosen from the selected group. The first step is to choose a particular drug group. For each cell the value given is multiplied by the weight to get a final value. The values under the four criteria are added together for each group of drugs. The group with the highest total is the selected group. The same process is then followed to select a particular P-drug from the chosen group. The next step is to verify the suitability of the selected P-drug for a particular patient. This is carried out according to the method described in the ‘Guide to good prescribing’ [9]. Then the students write the prescription.

The exercise is carried out during the practical sessions in Pharmacology. The students are divided into groups of 7 or 8 students each. Each group includes students mainly from Nepal, India and Sri Lanka and of both genders. The students carry out the exercise in their groups using reference materials and textbooks available in the college and departmental library. Access to the internet and primary sources is limited but may be available. The students get around one and half hours to carry out the exercise and then present their findings. The presentation is followed by a discussion.

In the practical examination students get around one and half hours to select their P-drug, verify the suitability of the drug for an individual patient and write the prescription. The problem is followed by viva-voce and assessment. The exercise accounts for 15 of the 50 marks allotted for the practical examination. The students are permitted to bring textbooks and other reference books and refer to them during the exercise.

The exercise has been carried out for different semesters of students. We regularized the exercise and introduced practical assessment for the present fourth semester students (January 2005 batch). At present, these students are in the fifth semester. The Manipal College of Medical Sciences (MCOMS), Pokhara, Nepal mainly admits students from Nepal, India and Sri Lanka for the undergraduate medical (MBBS) course. A hybrid approach of didactic lectures and problem-stimulated learning (PSL) sessions is used for Pharmacology learning [11]. Pharmacology is taught during the first four semesters in an integrated manner with the other basic science subjects (Anatomy, Physiology, Biochemistry, Pathology, Microbiology and Community Medicine). The exercise on P-drug selection has been carried out for over two years in our institution. However, the students were assessed in the practical exercise of P-drug selection and individualizing the selected P-drug to a particular patient only recently.

The exercise on P-drug selection has the objective of promoting rational use of medicines by students in their future career as doctors. Student feedback on this important exercise and on ways to improve and strengthen it was not obtained till date. To overcome this lacuna the present study was carried out. The authors obtained student opinion about the learning sessions on P-drug selection and about the assessment examinations. Strengths and weaknesses of the sessions and suggestions for improvement were elicited.

## Methods

Student opinion about the exercise and assessment was obtained using focus group discussions (FGD). The FGDs were carried out during November 2006. The student respondents were explained the objectives of the study and were invited to participate. Written informed consent to participate was obtained from the student respondents. The language of discussion was English, the medium of instruction for the MBBS course. The focus groups were selected to represent both genders and the three main nationalities, Nepalese, Indians and Sri Lankans who constitute the fourth semester student body. A group of 12 students were selected for the FGDs. We planned to select seven females and five males. With regard to nationality, four Nepalese, five Indians and three Sri Lankans were chosen. The final distribution of students with regard to gender and nationality was two Nepalese male students, two Nepalese females, three Indian females, two Indian males, one Sri Lankan male and two Sri Lankan females. The twelve students were divided into two groups of six students each. Each group had respondents of both genders and the three nationalities. The students were explained the objective and the general rules for the FGD. The students had not participated in a FGD before and the facilitator concentrated on putting the respondents at ease. The facilitator had a broad listing of the points to be covered during the FGD but the students were allowed to bring up other points which they felt were of relevance.

The FGD was conducted in the afternoon in the Pharmacology practical laboratory and lasted for about two hours. The session was recorded on camera with the group sitting in a semicircle. There was a gap of four days between the first and second FGD. The group of students for the two sessions was different. The practical learning sessions on P-drug selection, the assessment examinations and the small group dynamics were the major points of the discussion. Student responses were analyzed qualitatively under the major headings of practical learning sessions, practical assessment examinations, small group dynamics and other points.

One of the investigators (PRS) transcribed the discussion and the transcripts of each session were shown to the students involved. Emphasis in intonation and emotional expressions were not included. The transcribing was done by the investigator in consultation with the subjects involved keeping in mind the specific subjects' general mode of expression. The transcripts were then looked at in detail by the investigators to identify emerging themes.

## Results

Important verbatim statements which the investigators felt well illustrated a theme were selected.

**Practical sessions on P-drug selection:**

P-drug selection during the pharmacology practical was carried out in small groups. Around 25% of respondents had problems regarding the concept of a P-drug. Whether the P-drug is for a disease or a patient was the problem. One respondent got the concept quite right.

*“P-drug first we choose it for a disease. Then you bring the next thing that is the patient. Yes, When you prescribe the drug you consider the patient.”* (A) [We are numbering the cited student opinions alphabetically for ease of discussing them].

The students generally first looked at the disease and then proceeded further with the process of selection. A student says,

*“We carry textbooks in. We already have a general idea and we study the disease and then we rank the four criteria-efficacy, safety, cost and convenience. We usually start with efficacy. Most textbooks mention how efficacious a drug is. They also give the half-life etc. based on that we judge the efficacy.”* (B)

The student groups first selected a drug group and then a particular drug within the group. Group consensus or majority vote was often used to give the values. Students faced problems in ranking the drug groups. Some student groups did a relative comparison of the drug groups as a whole while others considered prototype drugs within each group. Calculating cost of drug groups was sometimes difficult. Coming to individual drugs, the large number of brands available in South Asia and the variation in cost among brands was a major problem.

Giving numerical values for the criteria was sometimes difficult. The groups adopted different approaches to solve the problem. Relative comparison between the drugs was emphasized. One group's approach is highlighted here.

*”We give values to the worst drug first, the drug we would never prescribe, say we pick it up. We give it a value according to the criteria; should be the minimum value. Then we start ascending on the values for the better drugs.”* (C)

The students felt that they lacked good reference sources. Some felt that access to the internet might be helpful while others had doubts about the quality of internet information. A respondent wanted the facilitators to select a few primary sources and other materials and distribute it to the different groups. The groups had problems finding out which drugs were available in Nepal. They usually relied on textbooks to select P-drugs. They felt that the skill of individualizing a P-drug to a particular patient was not emphasized.

**Practical assessment examinations on P-drug selection:**

The students wanted more time for the exercise in the practical examination. They wanted around two hours for P-drug selection, verifying the suitability of the P-drug for an individual patient and writing the prescription. The selection process during the practical classes was a team effort while during the examination the students have to carry out the exercise individually. They felt it was more difficult to select P-drugs for certain diseases.

*“Some diseases are extensively covered in the books. Like Malaria and all are so well covered. Some of them like scabies there is so little that to choose a P-drug becomes very difficult.”* (D)

Students usually bring their own textbooks during the examination and find them more convenient to use. The time factor makes it difficult for them to refer to more than two books. For drug prices, the students use Current Index of Medical Specialties (CIMS) or Drug Today. The viva-voce during assessment was felt to be helpful. The students got a chance to explain their scheme of P-drug selection to the examiner. The students felt that if the scheme of assessment is shared with them beforehand it will be very helpful.

Some students were strongly in favor of formative assessment of the group work during the practical sessions while others were more ambivalent. A student said,

*“Formative assessment should be done. Like someone works all the time, if our work is assessed in one final examination it is not a very fine thing. In the examination things can go wrong. It is matter of luck also.”*(E)

**Group dynamics during the PSL sessions:**

The students felt that the systematic random division of the groups (every sixth student being in a particular group according to roll number) was good. It ensured representation of all nationalities and both sexes. The groups were kept constant during a particular semester and this was appreciated. Some groups divided out responsibilities while others tackled the problem together. Absenteeism was a problem and some members did not get fully involved in the group activities.

The leadership role was usually assumed by the person who was going to present on a particular day. Presentation responsibilities were rotated. Some felt that the presenter should be randomly selected by the facilitators. The Sri Lankans sometimes brought a different perspective to the discussion compared to other nationalities. The respondents felt that personal problems and one-to-one relations between individual members did not affect group dynamics.

Coming to the gender roles, the boys felt that girls brought discipline to the group.

*“They may bring discipline in the group… yeah, that's true they are usually more hardworking and dedicated. We may go out of track sometimes but they bring discipline that I have to admit.”* (F)

The boys were felt to bring more practical knowledge and a sense of humor to the discussions. A girl said,

*“They explain things easily. Because sometimes girls find it difficult to explain things out. They put things so clearly and it becomes so easy to understand.”*(G)

The respondents were of the opinion that as far as possible a single boy or girl should not be put in a group.

**Other points:**

The students wanted the training to be continued during the clinical years and especially during the internship period. They felt it will be useful in their future clinical practice but had some reservations about how to actually apply it. They felt that the exercise has trained them to critically analyze information and will help them deal with aggressive pharmaceutical promotion. They wanted the exercise to be started earlier preferably from the second semester.

The physical environment of the pharmacology laboratory required improvement. The arrangement of desk, bench, desk, bench interfered with the group dynamics. It would be easier with tables and chairs. The students felt that in practice they would update their P-drug list about every six months. They were skeptical about new drugs and would wait for at least a year till new information became available before they would consider using it.

The student opinion regarding the practical sessions on P-drug selection and the assessment process of the skill during the pharmacology practical examination was positive. The group dynamics was also to their satisfaction. This was not explicitly stated by the respondents but was a point which emerged during the FGD. The demographic details of the students who participated are shown in Table 1.

**Table 1:** Demographic characteristics of focus group participants

Characteristic	Information
Training stage	Fourth semester medical students
Gender	5 males, 7 females
Nationality	4 Nepalese, 5 Indians, 3 Sri Lankans
Religion	7 Hindus, 4 Buddhist, 1 Christian

## Discussion

Problem-based learning of pharmacotherapy and the P-drug concept have been introduced in medical schools the world over. Problem-oriented pharmacotherapy teaching has been identified as a key intervention for promoting the more rational use of medicines [12]. Problem based learning of Pharmacology and Therapeutics has been carried out in medical schools all over the world [13], [14], [15].

The students may think that P(ersonal)- drugs are drugs personal to the patient, rather than personal to the doctor. P-drug is a drug ready for action and the dosage form, route and regimen are also required for a P-drug. The students initially only selected a P-drug but we were able to bring this concept across to the students in the later sessions.

**The cited student opinions:**

Opinion A: It has to be repeatedly emphasized that a P-drug is for a disease and not a patient. Being future doctors, medical students often bring in the patient right from the beginning. This has been mentioned in the Teacher's Guide to Good Prescribing [10]. A problem is that each disease has multiple variants and the student is confused about which variant to consider. We tell the students to consider the commonest presentation of a particular disease or condition.

Opinion B: Knowing where and how to find information may be as important in some cases as the information itself. There has been a change in emphasis from factual knowledge to concepts and ideas in medicine. Keeping this in mind, we decided to follow an ‘open book’ approach during P-drug selection and assessment. Efficacy is the most important criteria for P-drug selection. Often students have difficulty evaluating efficacy of drugs and head to head comparisons of drugs are usually missing in textbooks.

Opinion C: The process of giving relative weights to different groups of drugs and individual drugs is difficult. The grading is relative. One group adopted a practical approach to this rather difficult problem. They gave a value to the drug group which in their opinion was the worst. They then gave values to other drugs relative to the worst drug. A problem may be that sometimes, it may be difficult to decide which the worst drug group is.

Opinion D: The pharmacology textbooks used in our institution are either written by Western or Indian authors. The Nepalese books used are the Nepalese National Formulary and the Manual on Drugs and Therapeutics. These books are not updated frequently. The textbooks cover certain diseases in detail while others are not properly covered. Scabies is a common skin disease in Nepal but is not covered in detail in textbooks.

Opinion E: In our institution, assessment is carried during fortnightly tests, semester examinations and the University examinations. Sometimes, a student who has worked hard during the year may not do well in the assessment examination. We have started a system of formative assessment recently during the practical sessions.

Opinion F: The diversity of nationalities and of genders is a positive development. According to a male respondent, girls were more hardworking and dedicated and brought discipline to the group. This has been observed by the authors during certain group sessions but may not be true in all cases.

Opinion G: This was another opinion on gender roles this time by a female respondent. The female respondent was of the opinion that boys explain things clearly and make it easier for the group to approach and understand a problem.

**Problem faced during the sessions:**

Another problem faced by the students was inadequate knowledge about disease and the practical aspects of prescribing. During the first four semesters the emphasis is on the basic sciences and hospital visits are infrequent. The exercise on P-drug selection should be continued during the clinical semesters also. The large number of brands and the cost variation between brands especially in the Indian market is a major problem. Many medicines are imported into Nepal from India complicating the price scenario. We generally tell the students to consider the cost of the cheapest brand.

‘Teacher's guide to good prescribing’ says that students are not capable of identifying meaningful scores for the different criteria [10]. However, we found that the method developed by Joshi and Jayawickramarajah works quite well in practice. The emphasis on relative scores usually helped to arrive at realistic choices. There were occasional problems. We agree that students may not have developed a perception of the difference between a score of ‘6’ and a score of ‘7’. The relative grading is at the heart of the P-drug selection process.

The lack of good reference sources continues to be a big problem. Nepal has developed its own formulary and a Standard Treatment Schedule (STS) for health posts. However, due to various reasons, copies of the STS are not available in our institution. For the availability of drugs in Nepal, the Nepalese National Formulary (NNF) may be a good source. The last edition of NNF was however released in 1997. Many drugs have been licensed for use in Nepal in the last ten years. Nepal Drug Review (NDR) similar to CIMS is another good source for medicine prices and availability in Nepal.

**Individualizing the selected P-drugs:**

Individualizing the selected P-drug for a patient was a skill which the students felt was not emphasized. We plan to strengthen this aspect in the future. During the examination students have to work individually and they found this to be more difficult than the group work during the practical sessions.

**Learning to evaluate information:**

With the information explosion in medicine, learning to access information and to read efficiently are becoming important skills. We believe these sessions may be helpful for students to learn to evaluate information. This has to be confirmed by studying the same batch of students later in their careers. Allowing the students to bring reference sources during the examination would shift the emphasis from memory and factual recall to information analysis and critical appraisal. We agree that the students should be assessed during the examinations using a semi-structured check list and that the assessment scheme should be made known to students.

**Formative assessment:**

Formative assessment during the learning sessions would help in ensuring more active participation of the students. We are actively considering this proposal. Lack of manpower could be a limiting factor. Also formative assessment is not widely practiced in our institution.

**Diverse nature of the groups:**

The multinational and diverse nature of the group was seen to facilitate learning. This point emerged during the FGD and during feedback obtained by the department regarding other sessions. The Nepalese students were the most familiar with the availability and cost of medicines in Nepal. They were also familiar with the mechanism for primary care delivery. Sri Lanka has a long history of effective primary healthcare and emphasizes the use of essential medicines and the Sri Lankan students may have brought this into the deliberation. The Indian students bring a perspective of drug use problems in their vast country. Students from other nations also bring different national perspectives. The groups select a P-drug keeping in mind the situation in Nepal. Absenteeism was a problem and not all team members put in their full effort. The physical infrastructure continues to be weak and we are working hard to improve it. The constancy of the small groups during the semester helped in the group dynamics.

**Future plans:**

The exercise should be carried out during the internship training. At present during internship the major emphasis is on diagnosis. We are trying hard to introduce a module on rational use of medicines during the internship training. A number of exercises are being carried out to teach students about pharmaceutical promotion. A problem we find is that the P-drug concept has remained limited to pharmacology and has not become popular among clinicians. Involvement of clinicians is vital if the P-drug concept is to succeed.

In South Asia, the concept of P-drugs is still in its infancy. A problem-based learning session for teachers in medical colleges has to be organized. More support from the WHO and from colleges with experience in problem-based pharmacotherapy teaching and the P-drug concept is vital.

Our study had many limitations. The information was collected only from a single semester of students using FGD. The groups were selected from different nationalities and both sexes. However, we cannot rule out selection bias. Some of the minor nationalities have not been represented. The students we felt were frank in their comments. However, the facilitator of the FGD was a pharmacology faculty member and this may have had an inhibitory effect.

### Conclusions

The sessions on P-drug selection were appreciated by the students. The assessment process required improvement. The students wanted sessions on P-drugs during the clinical years and internship training. Formative assessment can be considered. The physical infrastructure needs improvement. There were practical problems in certain aspects of the P-drug selection process. Practical prescribing skills should be more emphasized.
